# Which Model Better Fits the Role of Aire in the Establishment of Self-Tolerance: The Transcription Model or the Maturation Model?

**DOI:** 10.3389/fimmu.2013.00210

**Published:** 2013-07-22

**Authors:** Mitsuru Matsumoto, Yumiko Nishikawa, Hitoshi Nishijima, Junko Morimoto, Minoru Matsumoto, Yasuhiro Mouri

**Affiliations:** ^1^Division of Molecular Immunology, Institute for Enzyme Research, University of Tokushima, Tokushima, Japan

**Keywords:** autoimmunity, thymic epithelial cell, self-antigen, gene transcription, cell differentiation

## Abstract

The discovery of Aire-dependent transcriptional control of many tissue-restricted self-antigen (TRA) genes in thymic epithelial cells in the medulla (medullary thymic epithelial cells, mTECs) has raised the intriguing question of how the single *Aire* gene can influence the transcription of such a large number of TRA genes within mTECs. From a mechanistic viewpoint, there are two possible models to explain the function of Aire in this action. In the first model, TRAs are considered to be the direct target genes of Aire’s transcriptional activity. In this scenario, the lack of Aire protein within cells would result in the defective TRA gene expression, while the maturation program of mTECs would be unaffected in principle. The second model hypothesizes that Aire is necessary for the maturation program of mTECs. In this case, we assume that the mTEC compartment does not mature normally in the absence of Aire. If acquisition of the properties of TRA gene expression depends on the maturation status of mTECs, a defect of such an Aire-dependent maturation program in Aire-deficient mTECs can also result in impaired TRA gene expression. In this brief review, we will focus on these two contrasting models for the roles of Aire in controlling the expression of TRAs within mTECs.

The current prevailing view regarding the role of Aire in self-tolerance is that it is involved in the transcriptional control of many tissue-restricted self-antigen (TRA) genes in medullary thymic epithelial cells (mTECs) (1). In other words, TRAs are considered to be the direct target genes of Aire’s transcriptional activity (the transcription model) (2). This view was first suggested in a paper reporting that Aire-deficient mTECs showed dramatically lower expression of TRAs than wild-type mTECs (3). Since this landmark report, the transcription model has prompted many studies of Aire in an attempt to clarify how the single *Aire* gene can influence the transcription of such a large number of TRAs within mTECs (4–7). Unfortunately, to obtain a mechanistic insight into this interesting phenomenon, it has been necessary to employ cultured cells transfected with the *Aire* gene, because the fraction of naturally Aire-expressing mTECs in the thymic stroma is too small to work with. However, no appropriate cultured cell lines that could be used reliably in place of Aire-expressing mTECs *in vivo* have been available. Nonetheless, overexpression of Aire in cultured cells resulted in increased transcription of many TRAs, in accordance with the transcription model. However, it is important to pay more attention to the uniqueness of bona fide mTECs *in vivo*, which show characteristics very different from those of cultured cell lines; although several cell lines derived from the thymic stroma are available for both humans and mice, none of them show typical characteristics of mTECs such as high expression of FoxN1, MHC class II, and CD80, even though they are positive for cytokeratin and epithelial cell markers (e.g., keratin 5, EpCAM, and lectin UEA-1 binding). Most importantly, none of these cell lines express a reliable level of Aire at the protein level. However, large aspects of Aire’s action on TRA gene expression in the transcription model were deduced on the basis of the effects of lack of Aire expression in mTECs *in vivo* (i.e., the phenotypes of Aire-deficient mice) and the opposite effects of Aire overexpression in mTEC-“like” cells *in vitro* (i.e., in transfection studies). The reality is that there is still a fairly wide gap between these two experimental settings that needs to be bridged.

In comparison with the remarkable changes noted in the expression profiles of TRA genes in Aire-deficient mTECs, morphological alterations in the medullary components from Aire-deficient mice were not initially appreciated until Farr’s paper had appeared (8). This was the main reason why insufficient attention was paid initially to another proposed explanation for the reduced TRA gene expression in Aire-deficient mTECs: the maturation model (2). However, fairly recent detailed studies of Aire-deficient thymi have revealed several important aspects of the Aire-dependent differentiation programs of mTECs, such as increased numbers of mTECs with a globular cell shape (8, 9), contrasted with reduced numbers of terminally differentiated mTECs expressing involucrin, the latter being associated with reduced numbers of Hassall’s corpuscles (9, 10). Although not fully investigated, increased percentages of mTECs expressing high levels of CD80 (CD80^high^) is another suggested aspect of the Aire-dependent mTEC differentiation program (11–13). In this regard, it is noteworthy that the Aire-dependent mTEC differentiation program can be linked to the control of TRA gene expression, in which the role of Aire may be perceived from a different viewpoint. Given that acquisition of the properties of TRA gene expression depends on the maturation status of mTECs (14, 15), any defect in such an Aire-dependent maturation program could also account for defects of TRA gene expression in Aire-deficient mTECs. In such a case, TRA genes would not have to be the direct target of Aire. Instead, Aire-deficient mTECs would have defective TRA gene expression, because they are not fully differentiated to stage(s) where other undetermined transcriptional means for TRA gene expression beyond Aire become available and/or active (Figure 1). Having said so, the exact point in the differentiation process at which Aire-deficient mTECs are prevented from differentiating further still remains unclear. Investigation of this issue has been hampered by the current lack of suitable markers for the mTEC differentiation program: so far, CD80 and MHC class II remain the few that are available. Precise elucidation of the target gene(s) relevant to the progression of mTEC differentiation controlled by Aire is an essential task to support the maturation model.

**Figure 1:**
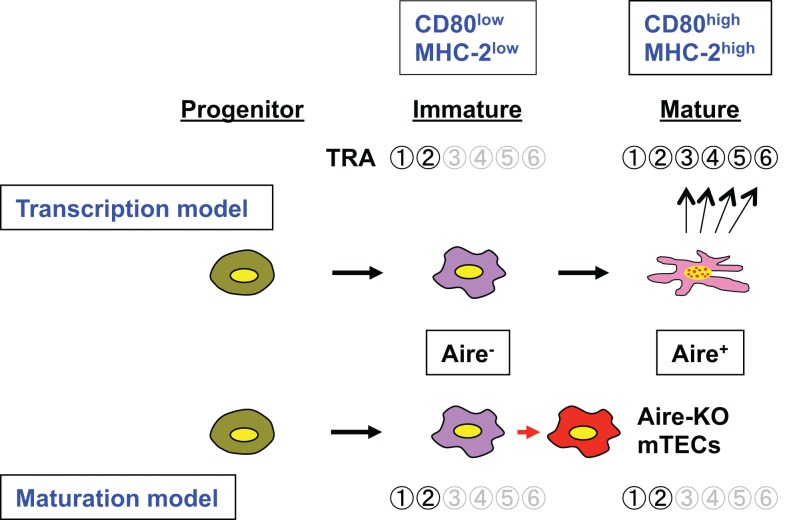
**Schematic representation of the roles of Aire in TRA gene expression associated with the mTEC differentiation program**. Aire-expressing mTECs develop from their progenitor via an immature stage (CD80^low^MHC-2^low^) where only limited numbers of TRAs (e.g., 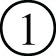 and 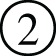) are expressed. Aire is expressed at the mature stage (CD80^high^MHC-2^high^), and the transcription model (upper) suggests that Aire directly activates many target TRA genes within mature mTECs (so-called Aire-dependent TRAs; 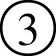
–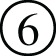). Consequently, lack of Aire results in defective expression of Aire-dependent TRA genes. In contrast, the maturation model suggests that Aire plays an important role in the maturation program of mTECs, and that expression of Aire-dependent TRA genes, such as 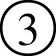
–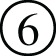, can be accomplished in terminally differentiated mTECs showing a dendritic to fibroblastic morphology that have fully matured with the help of Aire. According to this scenario, lack of Aire causes a halt in mTEC maturation at a stage where promiscuous gene expression in the cells is still not possible (marked as “Aire-KO mTECs”); lack of Aire in mTECs results in premature termination of differentiation, and these CD80^high^ Aire-less mTECs have a more globular cell shape and lack the transcriptional machinery for Aire-dependent TRA genes (lower). Because Aire-independent TRA genes, such as 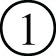 and 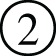, can be expressed before the mature stage, lack of Aire has little impact on their expression.

Currently, there is no firm evidence to support either model for Aire’s role in the control of TRA gene expression. Therefore, there is a need to develop better *in vivo* experimental systems for investigating this issue. In this connection, the results obtained by examining *Aire* gene expression under control of the rat insulin promoter using transgenic mice merit attention (16). It was found that alterations of the transcriptome did not mirror those created by abrogation of Aire within mTECs. This may not be surprising, but it is nevertheless important: if cell types differ, the effects of functional gain or loss of a transcription factor, such as Aire, can in turn differ markedly. This is especially important in the case of Aire, since mTECs are unique in showing promiscuous gene expression and a heterogeneous composition (17, 18). Thus, it cannot be over-emphasized that the roles of Aire need to be studied using *in vivo* models, and not *in vitro* systems using mTEC-surrogate cells.

Regardless of the models employed, there are several important issues related to the molecular regulation of TRAs within mTECs in the context of Aire. Obviously, the transcriptional control of TRAs is different from the regulation seen in their authentic tissues, as exemplified by differences in the transcriptional start sites of individual TRA genes (19). Although the fact that transcriptional hierarchies driving the development of the pancreas and transcription of the *insulin 2* gene are not maintained in mTECs (19, 20) might be more consistent with the transcription model, it does not contradict the maturation model in explaining why Aire-deficient mTECs show impaired *insulin 2* gene expression; the maturation model does not require the transcriptional hierarchies driving the development of authentic tissues. Instead, the maturation model has its own mTEC developmental process in which expression of particular TRAs is acquired at specific time points during the differentiation program. For example, expression of Aire-dependent TRA genes, such as *insulin 2* and *SAP1*, can be accomplished in terminally differentiated mTECs that have fully matured with the help of Aire protein. Lack of Aire in mTECs results in premature termination of differentiation, although Aire-deficient mTECs can still develop and pass a certain maturation stage. These Aire-less mTECs, which are rather mature (CD80^high^) but not fully competent for TRA expression, have a more globular cell shape and lack transcriptional machinery and/or activity for Aire-dependent TRA genes (9) (Figure 1). In this scenario, Aire-independent TRA genes, such as *CRP* and *GAD67*, can be normally expressed even in Aire-deficient mTECs, because these TRAs can be expressed before the terminal differentiation stage(s), and consequently the lack of Aire has little impact on their expression. It is still unknown why some (Aire-independent) TRAs are expressed from the immature stage(s), whereas (Aire-dependent) others are expressed only after they become fully mature. For this reason, it would be important to clarify the exact timing of Aire expression during the course of mTEC differentiation (21). Nevertheless, promiscuous gene expression seems to be accomplished in terminally differentiated mTECs that have matured in the presence of Aire protein (the maturation model). Alternatively, reduced TRA gene expression could represent failure of heterogeneity in terms of TRA gene expression due to a halt in differentiation at a premature stage before heterogeneity of individual mTECs has occurred. In contrast, the transcription model may explain why some TRA genes are Aire-dependent and others are not, as outlined in the following. Aire-PHD1 binding with H3K4me0 is an interesting finding, and a model has been proposed suggesting that Aire’s PHD1 acts as a chromatin reader, searching for genes showing low expression (5, 22); Aire preferentially binds with weakly expressed genes harboring the silent chromatin signature of H3K4me0, and may help to up-regulate TRA genes whose expression levels would otherwise remain low (23). In this model, TRAs showing relatively high expression would do not require the help of Aire, and their expression would be Aire-independent.

One caveat of the transcription model is that expression of Aire and Aire-dependent genes does not always overlap at the single-cell level (19, 24, 25). If Aire were controlling the expression of TRAs in any way by means of direct transcriptional control, we would expect to see both Aire and Aire-dependent TRAs within the same individual cell, although one could argue that the timing of expression of Aire and Aire-target genes might not always be the same within any given analytical snapshot time frame.

Involucrin is an interesting TRA in that it is sometimes used as an example of an Aire-dependent TRA that follows the transcription model: when cultured cell lines were introduced with an Aire-expressing plasmid, transcription of the endogenous *involucrin* gene was up-regulated possibly due to the transcriptional activity of Aire (26). However, at the same time, involucrin is also used as a marker of mTEC maturation status. Similarly to its expression in the epidermis of the skin, involucrin is expressed by mature epithelial cells in the thymic medulla; it is expressed most strongly in Hassall’s corpuscles, which seem to be the product of terminally differentiated mTECs, and Aire-deficient thymi show reduced numbers of involucrin-expressing mTECs, even in Hassall’s corpuscles (9, 10). These findings are consistent with data favoring the maturation model derived from *in vivo* systems suggesting that Aire is required for promotion of the mTEC maturation program. Thus, there is a need to understand the types of effects that can be expected according to the experimental systems employed.

Regarding the role of Aire in the mTEC maturation program, an important issue that needs to be carefully investigated is whether or not Aire has proapoptotic activity. Introduction of Aire into cultured cells has been reported to result in apoptosis (11). Accordingly, increased MHC class II^high^/CD80^high^ mTECs seen in Aire-deficient mice was considered to explain the lack of Aire-mediated proapoptotic activity, because loss of Aire did not result in augmented proliferation of mTECs (11). Given that Aire plays an important role in the induction of a wide variety of TRAs, concomitant induction of apoptosis in Aire-expressing mTECs by Aire itself might be an effective way to promote cross-presentation, thereby facilitating negative selection (1). Once again, however, this attractive hypothesis needs to be investigated in more depth using *in vivo* experimental systems.

Finally, there is a need to discuss the implications of these two different models for the mechanisms underlying the defect of negative selection in Aire-deficient animals. In principle, the transcription model restricts the failure of negative selection in Aire-deficient mice to reduced expression of TRA gene products. In contrast, the maturation model suggests that Aire may affect the thymic microenvironment more globally than through simple control of TRA expression levels. Consequently, the maturation model allows for the possibility that regulation of TRA gene expression may not be the major defect of Aire-deficient mTECs responsible for impaired negative selection. Instead, other alterations in the function of mTECs lacking Aire might equally account for the defective negative selection in Aire-deficient mice. These changes could include processing and/or presentation of self-antigens within the mTECs (27), the process of thymocyte maturation (28), the process by which mature thymocytes are attracted to their proper location for negative selection by production of chemokines from mTECs (27, 29), control of cross-presentation through alteration of the relationship between BM-APCs and mTECs (30), and the balance between negative selection and regulatory T cell production (31). Furthermore, modification of microRNA-regulated TRA gene expression by Aire might represent another dimension in this field that warrants further investigation (32). All of the above issues may be largely clarified once the target genes of Aire have been determined using *in vivo* models. Thus, our current understanding of the fundamental function of Aire still seems to be in its infancy, and the proposal and evaluation of different models would doubtless lead to further advances in this fascinating field of research.

## Conflict of Interest Statement

The authors declare that the research was conducted in the absence of any commercial or financial relationships that could be construed as a potential conflict of interest.
